# Modulation Effects of Cordycepin on Voltage-Gated Sodium Channels in Rat Hippocampal CA1 Pyramidal Neurons in the Presence/Absence of Oxygen

**DOI:** 10.1155/2017/2459053

**Published:** 2017-10-31

**Authors:** Zhi-Bin Liu, Chao Liu, Bin Zeng, Li-Ping Huang, Li-Hua Yao

**Affiliations:** ^1^School of Sport Science, Jiangxi Science & Technology Normal University, Nanchang, Jiangxi 330013, China; ^2^School of Life Science, Jiangxi Science & Technology Normal University, Nanchang, Jiangxi 330013, China; ^3^School of Pharmacy, Jiangxi University of Traditional Chinese Medicine, Nanchang, Jiangxi 330004, China

## Abstract

Our previous study revealed that cordycepin features important neuroprotective effects against hypoxic insult by improvement of neuronal electrophysiological function. Modulation on voltage-gated sodium channel (VGSC) in CA1 neurons is the initial event during hypoxia/ischemia. However, no study comprehensively investigated cordycepin on VGSC. Hence, this study investigated modulation effects of cordycepin on VGSC not only in oxygen physiological conditions but also in acute oxygen deprivation injury conditions. Results revealed that cordycepin (80 *μ*M) reduced the amplitude of VGSC currents (*I*_Na_) (77.6% of control, *p* < 0.01) within 1 min of drug exposure coupled with a negative shift in steady-state inactivation and prolonged recovery time course from inactivation. Additionally, this mild reduction on the peak of *I*_Na_ induced by the pretreatment with cordycepin can attenuate and delay the following hypoxia causing rapid dramatic decrease in *I*_Na_ with no additive change in the voltage dependence of inactivation. As modulation on VGSC in CA1 neurons represents the initial event during ischemia, we propose that suppression effect of cordycepin on VGSC is an important neuronal protective mechanism that may enhance neuronal tolerance to acute oxygen deprivation and delay hypoxia-induced neuronal injuries.

## 1. Introduction

Voltage-gated sodium channel (VGSC) plays a significant role in neuronal functions of the central nervous system, which is responsible for initiation and propagation of the neuronal action potential. Thus, VGSC is critical in signal communication between neurons and participates in regulating various physiological functions [1, 2]. In addition to its involvement in normal physiological events, increasing evidence suggests that VGSC also plays a key role in regulating pathophysiological processes, such as hypoxia [1–5]. Inhibition of VGSC activation results in reduced neuronal activity and Na^+^ influx across neuronal membrane, which in turn reduces metabolic demand on neurons in cases when energy production is severely compromised [2, 3]. This process ultimately increases neuronal tolerance to low-oxygen environments. Thus, inhibition of *I*_Na_ is usually considered as a cellular protective mechanism during initial stages of hypoxia [2–5].

Therefore, compounds modulating VGSC are developed for neuroprotective treatments [2, 4, 5]. Recent reports from our laboratory focused on the development of novel pharmacological actions from traditional Chinese medicine natural products. Cordycepin (3-deoxyadenosine), a major functional component of *Cordyceps militaris* [6], exhibits a wide range of biological effects, including antitumor [7, 8], anti-inflammatory [9, 10], antidiabetic [11, 12], and antioxidant activities [13, 14]. Recent reports suggested that cordycepin features neuroprotective effects on neuronal damage caused by ischemia/reperfusion insult by reducing oxidative damage, increasing free radical scavenging activity, and preventing neuronal cell death [13–15].

Studies from our laboratory demonstrated that cordycepin can increase neuronal tolerance during hypoxia and delay hypoxia-induced membrane depolarization and that the mechanism to suppress the neuron activity is strongly involved [16, 17]. As inhibition of VGSC activation would result in reduced neuronal activity and Na^+^ influx across the neuronal membrane, activation of VGSC plays a critical role in mediating sustained Na^+^ entry during ischemia and hypoxia, which then induce membrane depolarization [3, 18]. Thus, blocking these channels may exert neuroprotection during hypoxia [4, 5]. Although our previous study discovered that cordycepin selectively regulates activities of whole-cell Na^+^ current (*I*_Na_), no study comprehensively investigated its regulating mechanism [19]. Hence, in this study, the effect of cordycepin on the kinetics of VGSC in the hippocampal CA1 pyramidal neurons was investigated by using whole-cell patch-clamp techniques under voltage-clamp configuration [2]. Finally, actions of cordycepin on VGSC were also evaluated under hypoxia by using an energy-deprived injury model [16, 20].

## 2. Material and Methods

### 2.1. Drug Preparation

Chemicals used for making artificial cerebrospinal fluid (ACSF), tetrodotoxin (TTX), tetraethylammonium chloride (TEA-Cl), 4-aminopyridine (4-AP), Na_2_-ATP, CsCl_2_, ethylene glycol tetraacetic acid (EGTA), CdCl_2_, and hydroxyethyl piperazineethanesulfonic acid (HEPES) were purchased from Sigma Co. (St. Louis, MO, USA). Cordycepin with 98% purity was provided by South China Normal University [16, 21].

### 2.2. Preparation of Hippocampal Brain Slices

Animal studies were approved by the Institutional Care and Use Committee of Jiangxi Science and Technology Normal University. All experiments were performed on CA1 pyramidal neurons of hippocampal brain slices prepared from 15 to 22-day-old Sprague-Dawley rats as described in our previous studies [16, 22]. Animals were anesthetized with isoflurane and decapitated. The brains were quickly removed from the cranial cavity and immersed in ice-cold (4°C) oxygenated (95% O_2_/5% CO_2_) ACSF containing the following (in mM): NaCl 117, KCl 4.7, MgCl_2_ 1.2, NaH_2_PO_4_ 1.2, NaHCO_3_ 25, CaCl_2_ 2.5, and D-glucose 10 (pH 7.4). Osmolarity of bathing solution was adjusted to 325–330 mOsm with sucrose. Hippocampus was dissected free, and transverse hippocampal slices (400 *μ*m in thickness) were obtained using a vibrating microtome (NVSLM1, World Precision Instruments, USA). Slices were allowed to recover in continuously oxygenated ACSF for at least 1 h prior to experiments.

### 2.3. Patch-Clamp Recording

After recovery, individual slices were transferred to a recording chamber, which was continually perfused with oxygenated ACSF (unless otherwise indicated) at a rate of 4 ml/min. All experiments were performed at room temperature (26°C). Hypoxia was induced by switching from oxygenated ACSF equilibrated with 95% O_2_/5% CO_2_ to the same bath solution equilibrated with 95% N_2_/5% CO_2_ for >2 h. Bath solution was exchanged within ~20 s.

The following experiments were performed using conventional whole-cell patch recording under voltage-clamp configuration. Currents through VGSC were measured using a MultiClamp 700B patch-clamp amplifier (Axon Instruments, USA). Recording electrodes were fabricated from borosilicate glass pipettes (Sutter Instruments, USA) by a Flaming-Brown puller (P-97, Sutter Instruments, USA) and were filled with intracellular solution containing (in mM) CsCl_2_ 140, TEA-Cl 5, ATP-Na_2_ 2, EGTA 10, and HEPES 10 (pH 7.2) [19]. Electrode resistance reached 4–6 MΩ when pipettes were filled with solution.

Bathing solution was supplemented with 25 mM TEA-Cl, 5 mM 4-AP, and 0.4 mM CdCl_2_ to block delayed rectified K^+^ channels, transient outward K^+^ channels, and all Ca^2+^ channels, respectively [2, 19]. Recorded neuronal cells were allowed to stabilize for 1-2 min till the stable conditions. Cordycepin was dissolved in ACSF at concentrations of 20, 40, 80, and 200 *μ*M, and its effects were tested by bath perfusion (solution exchange was completed in about 20 s).

### 2.4. Data Analysis

Data were acquired by Clampex 10.5 via a digidata 1322 series A/D (Axon Instruments, USA) board at a sampling frequency of 20 kHz. Series resistance (10–20 MΩ) was monitored during the recording, and cells with changes > 30% in the series resistance were abandoned. Only one CA1 pyramidal neuron was tested in a hippocampal slice after the successful recording was made. Therefore, the number of samples (*n*) in each test group represents the cells recorded from different hippocampal slices.

Electrophysiological parameters were measured as previously described [2, 19]. For current-voltage activation curve plot, the current amplitudes before and after the cordycepin application were all expressed as percentages of the maximum current amplitude recorded initially under control conditions. For inactivation plots, the currents were normalized to their corresponding maximum values before and after cordycepin application. For recovery from inactivation plots, the recovery current amplitude (normalized with respect to the precondition pulse induced the current amplitude) was plotted versus times before and after cordycepin application. Steady-state activation kinetics of *I*_Na_ were obtained using a Boltzmann fit equation *G*/*G*_max_ = 1/(1 + exp((*V*_1/2_ − *V*_m_)/*V*_c_)), where *G* represents conductance at each command voltage, *G*_max_ refers to maximal conductance, *V*_m_ corresponds to command voltage, *V*_1/2_ denotes half-maximal activation, and *V*_c_ is proportional to the slope at *V*_1/2_. Steady-state inactivation of *I*_Na_ was obtained with another Boltzmann fit equation *I*/*I*_max_ = 1/(1 + exp((*V*_m_ − *V*_1/2_)/*V*_c_)), where *I*_max_ refers to maximal current amplitude, *I* represents the current amplitude measured from each command voltage, *V*_m_ corresponds to conditioning voltage, *V*_1/2_ denotes half-maximal inactivation, and *V*_c_ is proportional to the slope at *V*_1/2_. The time-to-peak value was used to analyze activation kinetics. Inactivation and deactivation time constants were obtained by fitting the current traces monoexponentially.

Results were presented as mean ± SEM. Statistical significance of difference was calculated using two-tailed Student's *t*-test. *p* < 0.05 level of confidence was considered statistically significant.

## 3. Results

### 3.1. Cordycepin Inhibited *I*_Na_ in a Concentration-Dependent Manner

**Figure 1:**
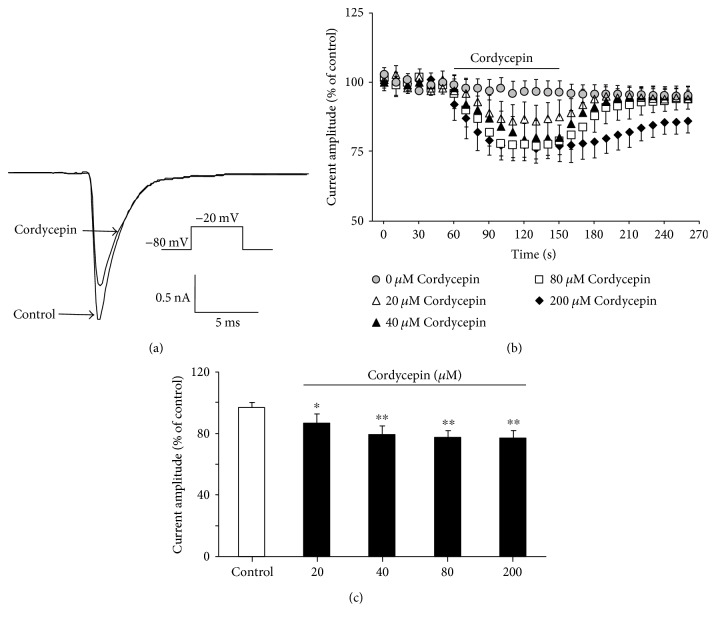
Cordycepin suppressed the amplitude of *I*_Na_ in a concentration-dependent manner in rat hippocampal CA1 pyramidal neurons. (a) Representative traces of evoked *I*_Na_ before and after 80 *μ*M cordycepin application in a whole-cell patch-clamp configuration. (b) Time courses of the effects of different concentrations of cordycepin (0, 20, 40, 80, and 200 *μ*M) on *I*_Na_ amplitude. Note that the bath application of cordycepin is indicated by a horizontal bar. (b) Effects of different concentrations of cordycepin (20, 40, 80, and 200 *μ*M) on *I*_Na_. The amplitude of *I*_Na_ after cordycepin application was expressed as a percentage of *I*_Na_ amplitude before cordycepin application. ^∗^*p* < 0.05, ^∗∗^*p* < 0.01 as compared with the control group.

*I*_Na_ was activated by using a step depolarization test pulse with a 50 ms duration from a holding membrane potential of −80 mV to −20 mV. In these experiments, *I*_Na_ was recorded at 10 s intervals. When *I*_Na_ reached a stable maximum amplitude, cordycepin was applied by bath perfusion. As shown in Figure 1, the amplitude of *I*_Na_ decreased in the presence of 80 *μ*M cordycepin (77.6% ± 4.58% of the control, *n* = 10; *p* < 0.01). After cordycepin (80 *μ*M) reached the chamber, inhibitory effects on *I*_Na_ occurred immediately and reached the maximum and stable value within 1 min, coinciding with our previous study [19]. Inhibition of cordycepin on the *I*_Na_ was concentration dependent (Figures 1(b) and 1(c)). As concentration of 80 *μ*M cordycepin caused maximal effects and can be washed out quickly (Figures 1(b) and 1(c)), this concentration was adopted for subsequent tests.

### 3.2. Effects of Cordycepin on *I*_Na_ Steady-State Activation

**Figure 2:**
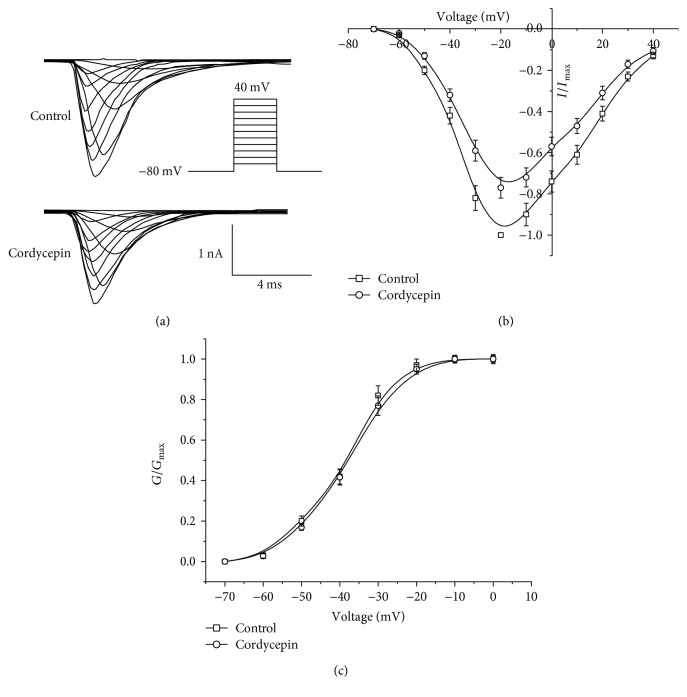
Effects of cordycepin on *I*_Na_ steady-state activation. (a) Representative traces of evoked *I*_Na_ before (top traces) and after (bottom traces) 80 *μ*M cordycepin application. (b) Current-voltage relationships of *I*_Na_ before and after 80 *μ*M cordycepin application. (c) Comparison of steady-state activation of *I*_Na_ before and after the 80 *μ*M cordycepin application.

From a holding potential of −80 mV, active currents were evoked by a series of +10 mV voltage steps to potential with 50 ms duration between −80 and +40 mV to test the effect of cordycepin on *I*_Na_ steady-state activation.  Figure 2(a) shows representative raw traces from both control and 80 *μ*M cordycepin recordings.

As shown in Figure 2(b), threshold for activation of *I*_Na_ measured −60 mV, and the amplitude of *I*_Na_ was maximal at −20 mV in the control and cordycepin-treated groups. The amplitude after cordycepin treatment was significantly lower than that of control at most voltage points (Figure 2(b)). However, steady-state activation curves for *I*_Na_ in the control (*n* = 10) and cordycepin (*n* = 10) treatment groups did not show a significant shift, as shown in Figure 2(c) (*p* > 0.05).

### 3.3. Effect of Cordycepin on *I*_Na_ Steady-State Inactivation

**Figure 3:**
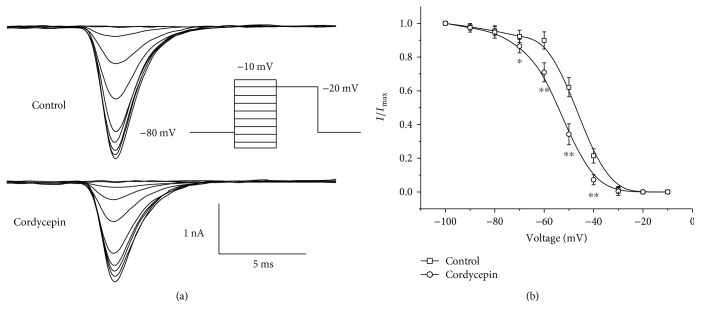
Effects of cordycepin on *I*_Na_ steady-state inactivation. (a) Current responses before (top traces) and after (bottom traces) 80 *μ*M cordycepin application examined with a dual-pulse protocols. (b) Comparison of steady-state inactivation of *I*_Na_ before and after the 80 *μ*M cordycepin application. ^∗^*p* < 0.05, ^∗∗^*p* < 0.01 as compared with the control group.

Figure 3(a) illustrates effects of cordycepin on voltage dependence of steady-state inactivation after examination with a dual-pulse protocol. Membrane potential was conditioned to different potentials (from −100 mV to −10 mV, with +10 mV increment) for 50 ms and then depolarized to a fixed test potential of −20 mV. Figure 3(a) displays representative *I*_Na_ traces before and after cordycepin treatment.  Figure 3(b) presents comparison of inactivation curves before and after cordycepin treatment. The figure shows a significant shift in steady-state inactivation curves in the control and cordycepin treatment groups (control: *V*_1/2_ = −47.4 ± 3.7 mV, *n* = 10; cordycepin: −54.8 ± 4.1, *n* = 10; *p* < 0.05). Application of cordycepin produced a 7.4 mV negative shift in the inactivation curve.

### 3.4. Effect of Cordycepin on *I*_Na_ Recovery

**Figure 4:**
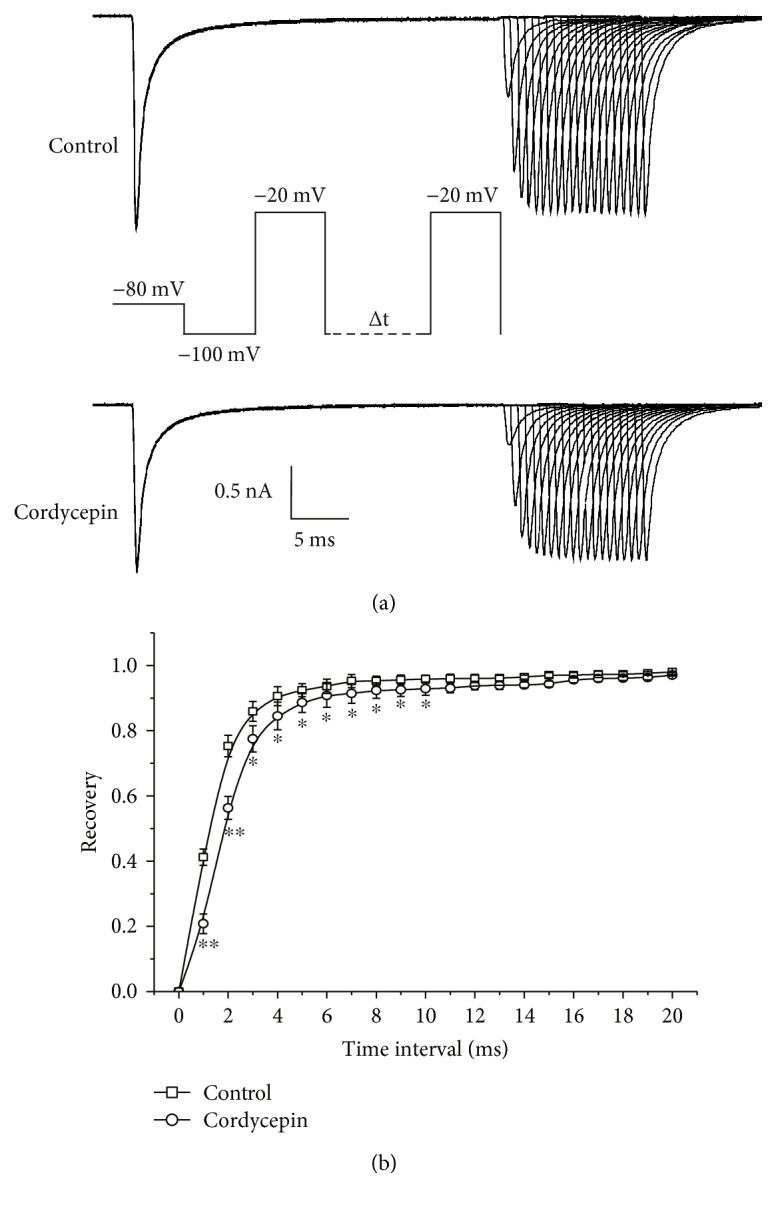
Effects of cordycepin on *I*_Na_ recovery from inactivation. (a) Representative traces of *I*_Na_ inactivation recovery before (top traces) and after (bottom traces) 80 *μ*M cordycepin application examined with a dual-pulse protocols. (b) Time courses of *I*_Na_ recovery from inactivation. ^∗^*p* < 0.05, ^∗∗^*p* < 0.01 as compared with the control group.

Recovery time course of *I*_Na_ from inactivation was investigated using a dual-pulse protocol (Figure 4(a)). A conditioning step (50 ms) from −100 mV to −20 mV was first employed to completely inactivate *I*_Na_. Then, after recovery at −100 mV for 1–20 ms, a test pulse of −20 mV was subsequently applied. Notably, after prolonged recovery (with recovery time from 1 ms to 20 ms) at −100 mV, the amplitude of *I*_Na_ gradually returned to control value (Figures 4(a) and 4(b)).  Figure 4(b) presents comparison of percentages of peak current recovery from inactivation before and after cordycepin application. Recovery current amplitude (normalized with respect to precondition pulse induced the current amplitude) was plotted versus times before and after cordycepin application. Recovery time course from inactivation was well fitted by a single exponential function, with a recovery time constant of 1.48 ± 0.06 and 2.10 ± 0.14 ms in the control (*n* = 10) and cordycepin (*n* = 10) (*p* < 0.05) groups, respectively. Cordycepin significantly reduced the rate of *I*_Na_ recovery from inactivation. These results indicated that *I*_Na_ in the cordycepin-treated group recovered from inactivation more slowly than those in the control.

### 3.5. Preapplication of Cordycepin-Induced Mild Inhibition on *I*_Na_, Attenuating and Delaying the Subsequent Hypoxia-Induced Rapid Dramatic Inhibition of *I*_Na_

**Figure 5:**
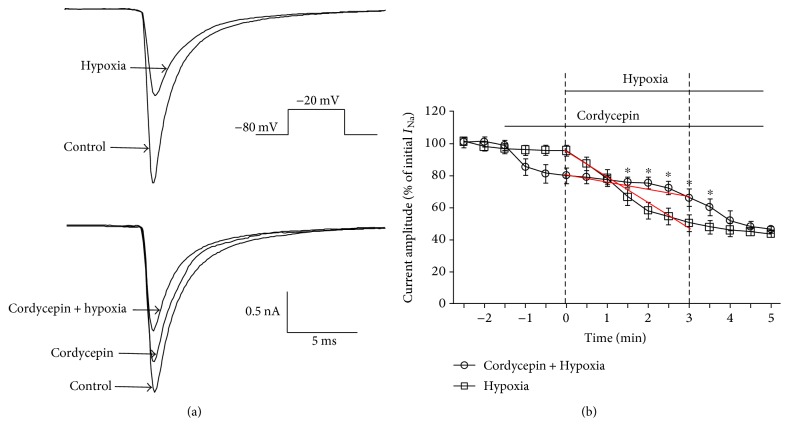
Preapplication of cordycepin-induced mild inhibition on *I*_Na_ attenuated and delayed the subsequent hypoxia-induced rapid dramatic inhibition of *I*_Na_. (a) Hypoxia for 3 min induced dramatic inhibition on the traces of *I*_Na_ (top traces) in the absence of cordycepin. Reduction on *I*_Na_ induced by hypoxia was attenuated in the presence of cordycepin (bottom traces). (b) Comparison of suppression effects of hypoxia on *I*_Na_ in the absence and presence of cordycepin. The descending slope was indicated by the red line. Hypoxia-induced rapid dramatic reduction on *I*_Na_ was attenuated and delayed in the cordycepin pretreatment group. ^∗^*p* < 0.05 as compared with the cordycepin pretreatment group.

Studies demonstrated that hypoxia can induce rapid dramatic inhibition on Na^+^ channels and current during initial stages (1–3 min) of hypoxia [3], which in turn resist the cell depolarization and reduce neuronal activity. This process will increase neuronal tolerance to low-oxygen environments [3, 23], indicating that there has been a self-adaptive cellular protective mechanism during initial stages of hypoxia. Thus, significant information can be obtained by investigating inhibition effects of cordycepin on *I*_Na_ during hypoxia, as account for neuroprotection effect of cordycepin from hypoxia insult [13, 15, 16, 24]. Like the previous studies reported [3], there was a rapid dramatic inhibition of peak *I*_Na_ when the extracellular bath was changed from control perfusate to the hypoxic solution. After 3 min of hypoxic exposure, *I*_Na_ reduced to 50.6% ± 5.12% of the baseline (*n* = 12; Figures 5(a) and 5(b), Table 1; *p* < 0.01). Steady-state inactivation was shifted by −9.2 ± 0.8 mV, and recovery time from inactivation also increased (recovery time constant in control: 1.51 ± 0.06 ms, *n* = 12; hypoxia: 2.21 ± 0.12 ms, *n* = 12; *p* < 0.05). Interestingly, response of *I*_Na_ to hypoxia was markedly blocked with cordycepin after exposure to hypoxia for 3 min (Figures 5(a) and 5(b), Table 1) although mild inhibition on *I*_Na_ was observed after pretreatment with cordycepin (Figures 5(a) and 5(b), Table 1). When neurons were exposed to hypoxia for 3 min with cordycepin pretreatment, hypoxia-induced inhibition of *I*_Na_ was significantly attenuated (66.3% ± 5.53% of initial *I*_Na_; *n* = 12; Figures 5(a) and 5(b), Table 1) compared with hypoxia only (50.6% ± 5.12% of initial *I*_Na_; *n* = 12; Figures 5(a) and 5(b), Table 1; *p* < 0.05). In the cordycepin pretreatment group, the descending slope (4.6 ± 0.32 mV/min, *n* = 12; Figure 5(b), Table 1) between 0 and 3 min after hypoxia treatment was obviously decreased when compared with hypoxia only (15 ± 0.11 mV/min, *n* = 12; Figure 5(b), Table 1). And most notably, the onset time of hypoxia-induced rapid dramatic inhibition on peak *I*_Na_ was also delayed from 0 min to 3 min in the cordycepin pretreatment group (Figure 5(b)), indicating that the neuron physical fitness response to external low-oxygen environments was improved through regulating self-adaptive cellular protective mechanism. No additive effects of hypoxia on the shift in steady-state inactivation and the time course of recovery from inactivation were observed (Table 1). These results indicated that mild inhibitory effect of cordycepin on *I*_Na_ channel may contribute to its neuroprotective effect against hypoxia insult.

## 4. Discussion

In the present study, we observed that cordycepin decreased the amplitude of *I*_Na_ in a concentration-dependent manner (Figure 1). Steady-state inactivation curves of *I*_Na_ shifted to more negative potentials (Figure 3), and time of *I*_Na_ recovery from inactivation was prolonged significantly by cordycepin (Figure 4). A negative shift on inactivation curve indicates low membrane potential threshold required for closing these channels. Slower recovery from inactivation implies prolonged transition of VGSC in cordycepin from inactivated to closed state and reduced fraction of available VGSC during spike trains [2]. These results imply that suppression of *I*_Na_ by cordycepin may inhibit intrinsic bursting and thus lead to a reduction in neuronal activity in CA1 neurons. This speculation was also confirmed by our previous study, which indicated that cordycepin can inhibit neuronal activity with low-frequency action potential bursting [17]. Furthermore, cordycepin pretreatment can significantly attenuate and delay hypoxia-induced rapid dramatic inhibition on *I*_Na_ (Figure 5, Table 1) with no additional effects on shifts in steady-state inactivation and recovery time course from inactivation (Table 1). This result indicates that suppression effect of cordycepin on *I*_Na_ and *I*_Na_ kinetics may contribute to its neuroprotection from hypoxic insult.

*I*_Na_ is responsible for both action potential generation and propagation and therefore plays a crucial role in neuronal excitability [1, 2, 25]. Thus, *I*_Na_ modulation may possess biological significance. Previous studies suggested that influx of Na^+^ contributes to brain damage during ischemia insult, as through activation of VGSC, Na^+^ influx across neuronal membrane mediates sustained Na^+^ entry, which in turn induces excessive membrane depolarization [2–4, 18, 25]. Consistently, evidence confirmed that excessive membrane depolarization may result from acute hypoxic or ischemic insults [3, 16, 25, 26]. Hence, inhibition of Na^+^ channel activation would reduce neuronal activity and reduce Na^+^ ion influx across neuronal membrane, which in turn against the hypoxia or ischemic induced the excessive membrane depolarization. Dong and Xu reported that mild inhibition in VGSC prolongs the duration, increases the threshold of excitation, and delays appearance of subsequent action potential, thus contributing to neuroprotection from hypoxic insult [2]. Other studies confirmed that reducing VGSC activity attenuates neuronal hypoxic responses and reduces hypoxia-induced neuronal injury and death in vitro and in vivo [2–5]. In the present study, we discovered that the application of cordycepin mildly inhibits VGSC (Figure 1), and it is coupled with a negative shift in steady-state inactivation (Figure 3) and slow time course of recovery from inactivation (Figure 4). Thus, we propose that cordycepin inhibition of VGSC may be an important mechanism to reduce neuronal activity, which in turn contributes to its neuroprotective effects against ischemic insults reported in our previous study [16, 17].

Additionally, to some extent, inhibition of *I*_Na_ is considered as a self-adaptive cellular protective mechanism during initial stages of hypoxia [3–5, 27]. As inhibition of Na^+^ channel activation reduces neuronal activity, this phenomenon results in reduction in energy demand at a time when energy production is severely compromised. This process ultimately increases neuronal tolerance to low-oxygen environments. Consistent with these deductions, we also noted that oxygen deprivation (hypoxia) causes rapid dramatic inhibition (within 3 min) on the peak of *I*_Na_ with a negative shift in steady-state inactivation and prolonged recovery from inactivation (Figure 5). We also observed that cordycepin pretreatment can significantly attenuate and delay hypoxia-induced rapid dramatic inhibition on *I*_Na_ (Figure 5, Table 1) with no additive effects on the shift in steady-state inactivation and recovery time course from inactivation (Table 1). The descending slope was markedly decreased between 0 and 3 min hypoxia (Figure 5, Table 1), and the onset time of hypoxia-induced rapid dramatic inhibition on peak *I*_Na_ was delayed from 0 min to 3 min in the cordycepin pretreatment group (Figure 5(b)). These results demonstrate that preapplication of cordycepin-induced mild inhibition on *I*_Na_ attenuates and delays subsequent hypoxia-induced rapid dramatic inhibition of *I*_Na_, indicating that the neuron physical fitness response to external low-oxygen environments was improved through regulating self-adaptive cellular protective mechanism, which will ultimately increase neuronal tolerance to low-oxygen environments and thus save more rescue opportunities from further deterioration induced by hypoxia. However, further investigations are needed to clarify the underlying protective mechanism.

In conclusion, the present study revealed that cordycepin can reduce peak *I*_Na_ coupled with changes in voltage dependence of inactivation of *I*_Na_, and this mild reduction on *I*_Na_ attenuates and delays hypoxia-induced rapid dramatic decrease in the *I*_Na_. As modulation on *I*_Na_ in CA1 neurons occurs initially during ischemia, we propose that cordycepin-induced mild inhibition of *I*_Na_ is an important neuronal protective mechanism that may enhance neuronal tolerance to acute oxygen deprivation and delay hypoxia-induced neuronal injury.

## Figures and Tables

**Table 1:** Preapplication of cordycepin-induced mild inhibition on *I*_Na_ attenuated and delayed the subsequent hypoxia-induced rapid dramatic inhibition of *I*_Na_. (Recordings were collected after 3 min hypoxia.)

Groups	*I*_Na_ amplitude (% of initial)	Descending slope (mV/min)	Inactivation, *V*_1/2_ (mV)	Recovery time (ms)
Control (*n* = 10)	96.8 ± 3.8		−47.4 ± 3.7	1.48 ± 0.06
Cordycepin (*n* = 10)	77.6 ± 4.58^##^		−54.8 ± 4.1^#^	2.10 ± 0.14^#^
Hypoxia (*n* = 12)	50.6 ± 5.12^##^	15 ± 0.11	−55.9 ± 3.9^#^	2.21 ± 0.12^#^
Cordycepin + hypoxia (*n* = 12)	66.3 ± 5.53^∗^^,##^	4.6 ± 0.32^∗∗^	−54.3 ± 3.8^#^	2.26 ± 0.14^#^

Descending slope means the inhibition rate induced by hypoxia from 0 to 3 min; ^∗^*p* < 0.05 and ^∗∗^*p* < 0.01 compared to the hypoxia group; ^#^*p* < 0.05 and ^##^*p* < 0.01 compared to the control group.
